# Intraperitoneal rescue stem cell therapy in experimental NEC mitigates intestinal, pulmonary, and neuroinflammatory injury in mice

**DOI:** 10.1038/s41390-025-04599-5

**Published:** 2025-12-02

**Authors:** Krishna Manohar, Fikir M. Mesfin, Sharon Joseph, Jasmine Lee, Jianyun Liu, William Chris Shelley, John P. Brokaw, Troy A. Markel

**Affiliations:** 1 Department of Surgery, Indiana University School of Medicine (IUSM), Indianapolis, IN USA; 2 Riley Hospital for Children at Indiana University Health, Indianapolis, IN USA

## Abstract

**Background:**

Necrotizing enterocolitis (NEC) is a devastating condition associated with long-term complications including NDI (neurodevelopmental impairment), chronic lung disease, and intestinal complications. Umbilical-cord-derived mesenchymal stem cells (USCs) have been shown to improve intestinal outcomes when given prophylactically in preclinical NEC models. We hypothesized that intervention with USCs following onset of experimental NEC would also improve outcomes.

**Methods:**

NEC was induced in 5-day-old mouse pups through formula gavage, hypoxia, and hypothermia. Control pups were left with mother (BF ctrl, *n* = 21). Experimental animals were given an intraperitoneal (IP) injection of either USCs (NEC + USC, *n* = 16) or sterile PBS (NEC + PBS, *n* = 18) on PND(postnatal) 7. On PND 9, pups were euthanized, intestinal and lung tissues were H&E stained and homogenized for cytokine assessment, while brain microglia were analyzed via immunohistochemistry.

**Results:**

When compared to the NEC + PBS group, the NEC + USC group showed improved clinical scores, intestinal and lung histology, and an improved cytokine profile. Brain microglia were more characteristic of a resting state, with increased branching and complexity that was similar to breast-fed controls.

**Conclusions:**

These findings suggest that intraperitoneal USC administration after NEC onset improves clinical outcomes and reduces intestinal, pulmonary, and neuronal injury—highlighting its potential as a therapy after onset of experimental NEC.

**Impact:**

- Umbilical-cord-derived mesenchymal stem cells (USCs) can improve outcomes in NEC even after its onset.
- This study is the first murine model to show that USCs serve as an effective rescue therapy—demonstrating significant improvements in clinical scores, intestinal health, lung function, and brain microglia activity.
- The research suggests that IP USCs may reduce secondary organ damage and enhance long-term outcomes for affected infants, paving the way for future pre-clinical and clinical trials in the neonatal care of this devastating disease.

## Introduction

Necrotizing enterocolitis (NEC) is a severe intra-abdominal disease that disproportionately affects premature neonates and is associated with a high morbidity^1,2^ and mortality.^3^ This devastating disease is characterized by abdominal distension, intestinal inflammation, microbial translocation, systemic inflammation, and potential multiorgan failure and death.^4,5^ With the destruction brought forth by this disease, research into new and innovative therapies is imperative—with stem cell therapy emerging as a burgeoning therapeutic of interest. Specifically, mesenchymal stem cells (MSCs) have been studied extensively over the past 10 years as a potential treatment option in models of NEC,^6–9^ and have been shown in several pre-clinical studies to improve outcomes of NEC.^7,9–11^ These cells are multipotent cells that can differentiate into a variety of cell types^12,13^ and are suspected to reduce inflammation and rescue injured tissue^7,14–16^ through the release of vital paracrine mediators.^17^

In murine models of NEC, mouse pups that received intraperitoneal injection of stem cells *prior* to induction of NEC had lower incidences of NEC (and lower incidences of severe NEC), decreased intestinal permeability, and improved intestinal barrier function.^7,18,19^ Additionally, in adult murine models of intestinal ischemia/reperfusion injury, stem cell therapy has demonstrated improved survival, intestinal histology, increased intestinal perfusion, and the local downregulation of various proinflammatory cytokines.^20–22^

The clinical effects of NEC outside of the intestinal tract are well documented. Infants who survive NEC are known to have both short and long-term neurodevelopmental impairment^23–26^ as well as chronic lung disease.^27,28^ In terms of neurodevelopmental impairment, the inflammatory cascade of the intestine that is characteristic of NEC has been implicated in increased white matter injury and microglial activation. This phenotypically leads to a higher incidence of autism, cerebral palsy, cognitive deficits, and visual/hearing impairment.^23–26,29^ Similarly, the microbial dysbiosis and translocation that occurs in NEC causes systemic inflammation and activation of toll-like receptors in the lung that results in neonatal lung injury^27,28^ of varying severity. In fact, a multicenter cohort study from Spain found that preterm infants with surgical NEC had an odds ratio of 2.0 of developing bronchopulmonary dysplasia.^30^

Although there are no direct studies that look at the effects of stem cell therapy on these secondary deficits associated with NEC, there are studies that investigate how MSC therapy improves the pathologic effects of these disease processes.^31,32^ For example, MSC therapy has been shown to increase myelination and decrease neuroinflammation in a hypoxic-ischemic model.^29,33^ Intranasal administration of MSCs in rat pups with hypoxic-ischemic brain injury significantly reduced loss of brain tissue and markers of neuroinflammation.^34,35^ In terms of lung injury, rat models of bronchopulmonary dysplasia with MSC treatment have demonstrated improved lung compliance, preserved alveolar growth, restoration of alveolar architecture, and an increase in bronchioalveolar stem cells.^36–39^

We therefore sought to further study the effects of MSC therapy in a murine model of NEC, but to specifically look at the benefit as a “*rescue”* therapy (where the stem cells were given halfway through the NEC experimental protocol). We hypothesized that umbilical cord-derived umbilical mesenchymal stem cell (USC) therapy could still improve intestinal, neurological, and pulmonary sequelae in mice exposed to experimental NEC.

## Methods

### Animals

The Indiana University Institutional Animal Care and Use Committee (IACUC) oversaw and approved all animal protocols and use under protocol #19122. Wild-type C57Bl/6J mice were obtained (Jackson Laboratories, Bar Harbor, ME) and bred in-house at Indiana University. For the above experiments, individual pups in each litter were randomly assorted to each therapeutic group. This was done to minimize the effects of any litter-based differences. The results from multiple breeding cycles were pooled to obtain enough “n” for each group. Due to the early age and sexual immaturity of the pups at the time of the study (5–9 days old), sex was not considered as a confounding factor, although each clinical group had a mix of male and female pups.

### Necrotizing enterocolitis

NEC was induced in mice as previously described.^40–43^ In brief, pups were separated from their mothers on PND 5, randomly sorted into different therapy groups, and placed in an incubator with temperature set at 32 °C and humidity set at 40%. Control pups remained with mothers to breastfeed. Three groups were studied (*n* = 16–21) per group: (1) Breast fed control mice (BF ctrl), mice undergoing NEC with an intraperitoneal (IP) injection of phosphate-buffered saline (PBS) (NEC + PBS), and mice undergoing NEC with an IP injection of human-umbilical cord derived stem cells (ATCC, Washington, DC) given at a concentration of 80,000 cells per gram of weight (NEC + USC). IP injections were given on the morning of PND 7. Many models of NEC, including our own^41,42,44–46^ demonstrate clinical and histological changes of NEC by this time. Pups undergoing experimental NEC were gavage-fed hyperosmolar formula (300 kcal/kg/day) 3 times per day using a 1.9 French Catheter. The formula was prepared fresh every 48 h using Esbilac milk replacer (PetAg, Hampshire, IL) and Similac Advance powder (Similac, Columbus, OH). The formula was stored at 4 °C and warmed up in the incubator just prior to feeding. Lipopolysaccharide isolated from Escherichia Coli O111:B4 (cat # L4391, Sigma-Aldrich Company, St. Louis, MO) was added at 8 mg/kg daily to formula. Pups were exposed to hypoxia in a chamber with 5% oxygen for 10 min three times a day (before each feed) and to hypothermia at 4 °C for 12 min twice a day (in the morning and evening). Pups were assessed for clinical scores and weight gain. On PND 9, pups were euthanized via cervical decapitation, and tissues were harvested for further analysis. Mice who succumbed prior to reaching PND 9 were excluded from tissue analysis.

### Umbilical cord stem cell injection

The human umbilical cord-derived MSCs were sourced from ATCC (Washington, DC). The cells were cultured in house and for the purposes of these experiments, cells from a passage number between 8 and 12 were utilized.^20,21,47^ Cells were thawed and plated on flasks and allowed to grow for two full days. Early on the third day of the NEC protocol (corresponding with PND 7 in the mouse pups), cells were counted and resuspended in PBS. A total dose of 80,000 cells per gram^20,21,47^ was suspended in PBS, with a total volume of 10 µL per injection—which was given to pups via an IP injection using a Hamilton syringe and a 30-gauge needle. Care was taken to pass the needle subcutaneously near the liver and gradually towards the midline before puncturing the peritoneum to ensure successful delivery of the stem cells into the peritoneal cavity and prevent leakage. The syringe was thoroughly cleaned before and after each procedure, and the stem cell solution was drawn up in a manner that avoided air bubbles. The vehicle control for the experiment was an intraperitoneal injection of 10 μL of PBS.

### Clinical assessment

Clinical status was assessed on all experimental and breast-fed controls on PND 5 and 9. The scoring system has been used and validated in previous experiments in our lab.^40–43^ In brief, scores range from 0–12 and assess four categories (with subcategory scores ranging from 0–3), including: hydration status, activity, alertness, and body color.

### Evaluation of macroscopic intestinal damage

Upon euthanasia, the terminal ileum was collected, examined, and scored from 0 to 2 based on its consistency, color, and dilation, as previously detailed.^40–43^ The final score is reported as a sum of each category, with a maximum intestinal injury score of 6. For example, a score of 0 represented an intestinal segment that appeared normal with no discoloration or dilation. A sum score of 6 (with a sub-score of 2 in each category) represented an intestine that was very fragile, with a “jelly-like” consistency, and extensive discoloration/dilation/necrosis.

### Evaluation of microscopic intestinal damage

Samples from the terminal ileum were fixed in 4% paraformaldehyde for 24 h, dehydrated in 70% ethanol, embedded in paraffin, and sectioned/ stained with hematoxylin and eosin. 20× images of the intestine were taken throughout the sample, with an average of 6–8 images per pup. These images were randomly mixed, and scoring was performed blindly by two independent researchers and reported as an average of the two scores. The histological damage was scored on a scale from 0 to 4 as previously described.^40–43^ A score of “0” represented intestine with intact villus architecture and basement membrane, whereas a score of “4” represented frank loss of the architecture, bowel necrosis, and/or signs of perforation.

### Microglial analysis

A thin slice of the center of the mouse brain was cut in a coronal dimension, processed, and mounted onto a histological slide and stained with Iba-1—a microglial marker—for immunohistochemistry. This antibody (WAKO 019-19741-Fujifilm, Japan) is a polyclonal rabbit antibody with good cross-species reactivity with human and mouse microglia. Multiple 40x images were taken of these sections in the cortical region (an easily definable outer-most layer) of the cross-sectional slice of tissue. Microglia were analyzed using Image J software using a variation of a previously published protocol^43,48^ and as previously detailed by our lab.^43^ In brief, microglia with intact nuclei were isolated and analyzed using the *skeletonize* Image J plugin to characterize branching of the microglia, branch length, branch number, and the junctions of each branch. The second type of analysis involved outlining the microglia using the *FracLac* Image J plugin to characterize the microglial shape and to measure area, perimeter, and Iba-1% staining per cell to overall characterize the spread of the microglia. Higher branching and higher spread indicate a resting, ramified state of the microglia, while the reverse suggests an activated shape.^49,50^

### Evaluation of lung injury and terminal differentiation

At euthanasia, the trachea was cannulated and instilled with PBS until the lungs were inflated. The right lower lobe of each pup was harvested and fixed for 24 h in 4% paraformaldehyde. The tissue was then dehydrated with 70% ethanol, embedded in paraffin, sectioned, and H&E stained. The assessment of lung injury in neonatal mouse pups was conducted using two main parameters. The first parameter was alveolarization. This term refers to the last of the histological stages of lung development and differentiation of alveolar sacs, during which the majority of gas-exchange surface is formed.^51^ This process can be adversely affected by prematurity and inflammatory processes.^51,52^ We quantified this concept using the ratio of airspace over the alveolar surface area. A coronal slice of the whole lung was examined on the slide and photographed. The images were then randomized and coded to ensure blinding. Three to four 40X magnification snapshots of open airspaces at the periphery were captured of each lung, making sure to include only differentiated alveolar sacs and exclude bronchioles. ImageJ was used to select the total air space exposed to alveoli as well as to highlight the perimeter of the alveolar walls. A ratio of these measures was used to indicate the total alveolarization, with higher ratios indicating wide open-air spaces and poorly differentiated alveoli. Smaller ratios indicated well-differentiated alveoli with more alveoli to air space. The images were then decoded, and the ratios were averaged per experimental group. A second parameter used to assess lung injury was a scoring system adapted from the American Thoracic Surgery Society, and previously validated method from our lab.^45,46^ This system considers neutrophil infiltration (in alveoli and the interstitium), presence of hyaline membranes, alveolar septal thickening, the presence of red blood cells within the alveolar space, and/or proteinaceous debris. For this method, three 20X photos were obtained from each, and these images of lung histology sections were blindly assessed using this previously described scoring system. Briefly, each category listed above was scored from 0 to 2, with a total maximum score of 12, indicating severe lung injury.

### Tissue and serum cytokine analysis

The right lower lung lobe and segments of the terminal ileum of mouse pups were removed and placed in conical tubes with RIPA (radioimmunoprecipitation assay) buffer (Millipore Sigma, St. Louis, MO). Tissue was then homogenized using an ultrasonicator. Tubes were centrifuged at 10,000 × *g* for 5 min, and cell lysates were removed. Samples of blood were also taken from pups following cervical decapitation. These samples were spun down at 2800 g for 5 min, and the supernatant was separated out as serum samples. Cell lysates and serum supernatants were then used to measure cytokine levels via a Th17-multiplex beaded assay (EMD Millipore, Burlington, MA) and included IL-17A, IL-33, IL-6, TNF-α, IL-22, IL-10, and IL-1β. If no cytokine response was picked up in all of the samples, they were not included in the graphical representation of cytokine data for comparison in each compartment (intestine, lung, serum). These experiments were performed in duplicate to ensure accuracy and reproducibility. Of note, some samples from breast-fed controls that were run in parallel to the disease/treatment groups have also been published in a different study looking at a different therapeutic mechanism in a murine model of NEC.^42^

### Statistical analysis

GraphPad Prism 9 was used for statistical analysis. Data were assessed for normality using Shapiro-Wilk tests. Continuous and normally distributed data were expressed as mean ± standard deviation (SD). Ordinal data and non-normally distributed data were expressed as medians and interquartile ranges. Data were analyzed with t-test, Mann–Whitney U-test, Kruskal-Wallis, and ANOVA, where appropriate. For Kruskal-Wallis and ANOVA comparisons, if significance was found, ad-hoc comparisons were performed, and Dunn’s correction for multiple comparisons was performed. A *p* < 0.05 was considered statistically significant. Figures were created using GraphPad Prism 9.

## Results

### Stem cell rescue therapy improves outcomes in NEC experimental model

**Fig. 1:**
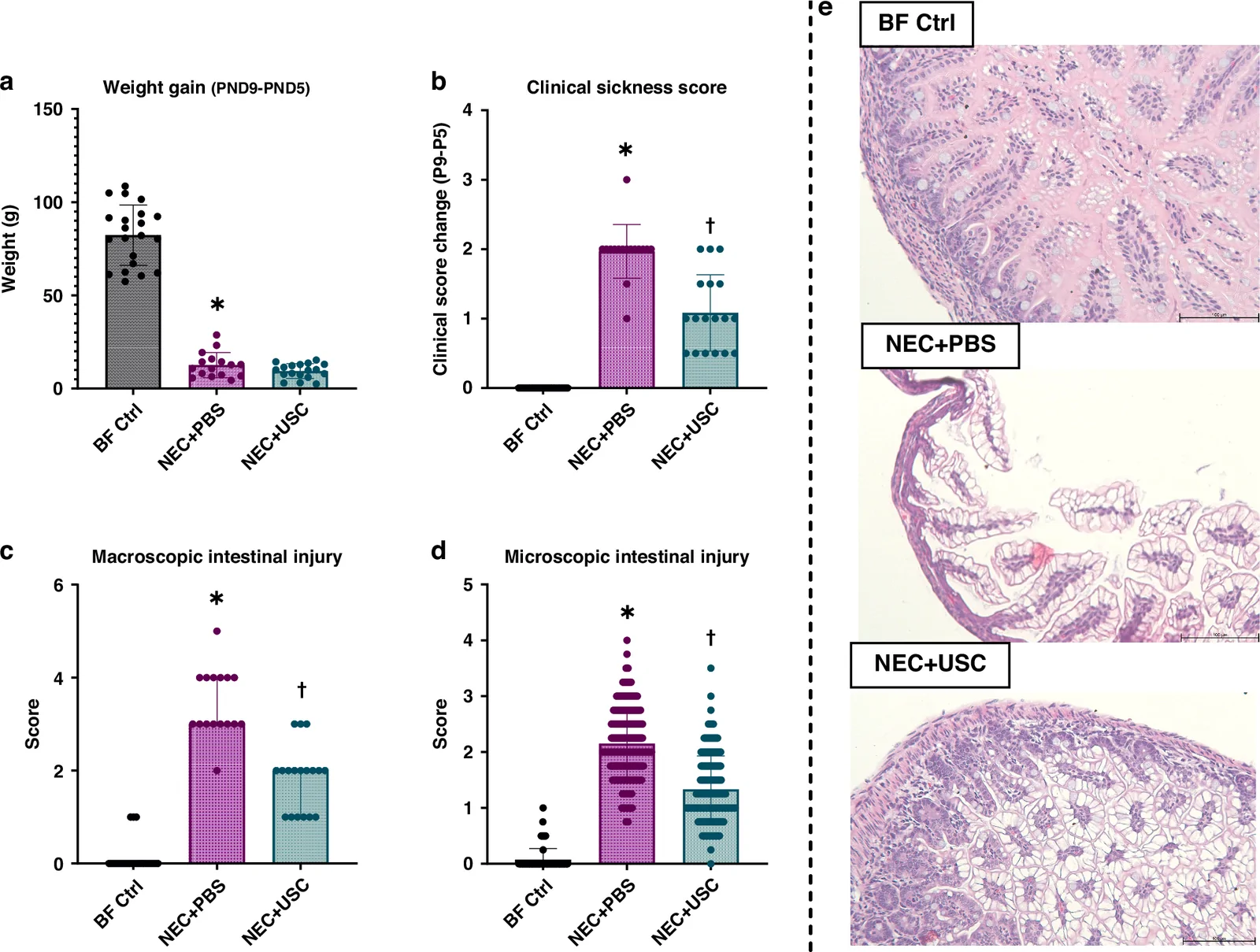
Stem cell rescue therapy improves clinical outcomes in NEC. The vehicle group (NEC + PBS) has worse (**b**) clinical sickness (*p* < 0.0001), **c** macroscopic (*p* < 0.0001)/ **d** histologic (*p* < 0.0001) intestinal injury scores when compared to breastfed controls (BF cTRLs). This is improved with the stem cell therapy group (NEC + USC) in (**b**) clinical sickness (*p* = 0.0258) and **c** macroscopic (*p* = 0.0009)/**d** histologic (*p* < 0.0001) intestinal injury scores. There were no differences in (**a**) weight gain. **e** Representative histological images (20X) of the intestines of breast-fed controls (BF ctrl), vehicle group (NEC + PBS), and the stem cell therapy group (NEC + USC) are shown with scale bars reflecting a distance of 100 µM. (*p < 0.05) Comparisons between breast-fed controls (BF ctrl) and vehicle controls (NEC + PBS), (†p<0.05) comparisons between vehicle group (NEC + PBS) and stem cell therapy groups (NEC + USC).

There was a significant decrease in average weight gain between breast-fed controls and NEC + PBS (*p* < 0.0001), but no improvement in weight gain seen in the stem cell therapy group(*p* = 0.6318) [BF ctrl: 82.3 ± 16.2; NEC + PBS: 12.6 ± 6.8; NEC + USC: 9.4 ± 4] (Fig. 1a). The NEC vehicle group (*n* = 16) had worse clinical sickness scores (*p* < 0.0001) when compared to the breast-fed controls (*n* = 21), and this was improved (*p* = 0.0258) in the stem cell treatment group(*n* = 18) when compared to the NEC vehicle group [BF ctrl: 0(0-0); NEC + PBS: 2(2–2), NEC + USC: 1(0.5–1.5)] (Fig. 1b). Additionally, there was a significant worsening (*p *< 0.0001) of the macroscopic histologic intestinal score in the NEC vehicle group compared to the breast-fed controls with improvement (*p* = 0.0009) seen in the stem cell therapy group [BF ctrl: 0(0-0); NEC + PBS: 3(3–4), NEC + USC: 2(1–2)] (Fig. 1c). Similarly, there was a significant worsening (*p* < 0.0001) of the microscopic (histological) intestinal score in the NEC vehicle group compared to the breast-fed controls with improvement (*p* < 0.0001) seen in the stem cell therapy group [BF ctrl: 0(0-0); NEC + PBS: 2(1.75–2.5), NEC + USC: 1.25(1–1.75)] (Fig. 1d). Representative images of microscopic intestinal histology are shown in Fig. 1e.

### Stem cell rescue therapy improves acute brain injury

**Fig. 2:**
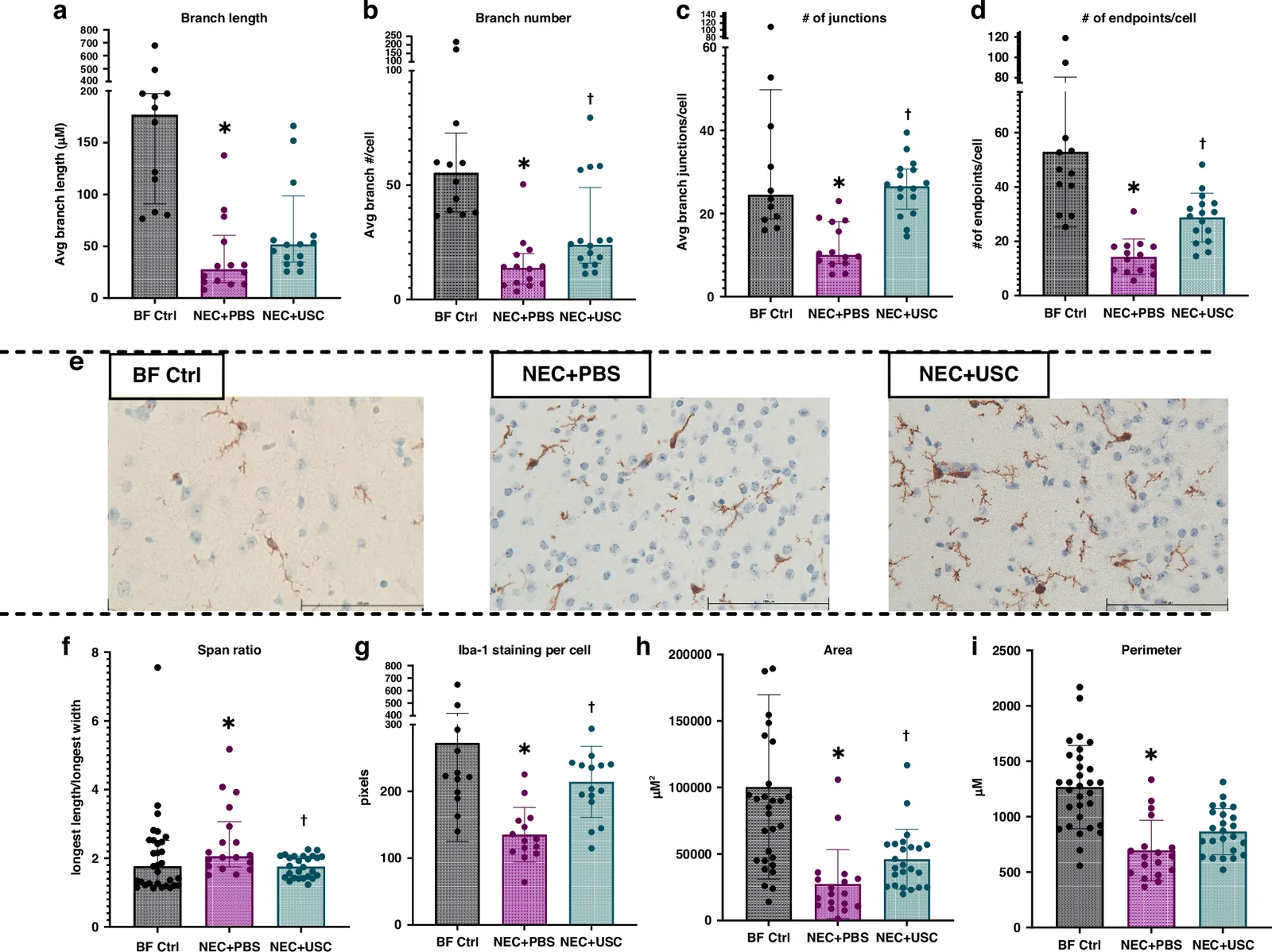
Stem cell rescue therapy prevents acute brain injury. Compared to brains of breast-fed controls, microglia of the vehicle group exhibit a more activated phenotype characterized by decreased (**a**) microglial branch length (*p* < 0.0001)/ (**b**) branch number (*p* < 0.0001)/ (**c**) branch junctions(*p* = 0.0002)/ **(d)** branch endpoints (*p* < 0.0001), an increased (**f**) span ratio (*p* = 0.0391), and decreased (**g**) Iba-1 staining per cell (*p* = 0.0004)/ (**h**) area(*p* < 0.0001)/ (**i**) perimeter (*p* < 0.0001). Compared to the microglia of the vehicle group, microglia in the brains of mice in the treatment group (NEC + USC) have increased average (**b**) branch number (*p* = 0.0329)/ (**c**) branch junctions(*p* < 0.0001)/ (**d**) branch endpoints (*p* = 0.0037),  a decreased (**e**) span ratio (*p* = 0.0356) and (**g**) increased microglial Iba-1 expression per cell (*p* = 0.001)/ (**h**) overall area/cell (*p* = 0.0007). (**e**) Representative histological images of microglia of the cortical region of breast-fed controls (BF ctrl), vehicle group (NEC + PBS), and the stem cell therapy group (NEC + USC) are shown with scale bars reflecting a distance of 100 µM (*p < 0.05) Comparisons between breast-fed controls (BF ctrl) and vehicle controls (NEC + PBS), (†*p* < 0.05) comparisons between vehicle group (NEC + PBS) and stem cell therapy groups (NEC + USC).

When compared to the brains of breast-fed controls, the brains of mice in the vehicle group (NEC + PBS), exhibited a more activated microglial phenotype with less branching and complexity as evidenced by an decreased average microglia branch length (*p* < 0.0001), branch number (*p* < 0.0001), branch junctions(*p* = 0.0002) and branch endpoints (*p* < 0.0001)(Fig. 2a–d). Additionally, in comparison to the breast-fed controls, the microglia of the vehicle group were more elongated and ameboid characteristic of an activated phenotype which is quantified by an increased span ratio (*p* = 0.0391) (Fig. 2f). Finally, these microglia demonstrated a decreased spread of their ramified processes as demonstrated by a decreased Iba-1 staining per cell (*p* = 0.0004), area (*p* < 0.0001), and perimeter(*p* < 0.0001) (Fig. 2g–i). The microglia in the brains of mice treated with USCs demonstrated signs of microglia in a more resting, ramified state when compared to the vehicle (NEC + PBS) group, with greater branching and complexity as evidenced by an increased average branch number (*p* = 0.0329), branch junctions(*p* < 0.0001), and branch endpoints (*p* = 0.0037) (Fig. 2b–d). These microglia were also less elongated as described by a decreased span ratio (*p* = 0.0356) (Fig. 2f). In addition, these microglia demonstrated a greater spread of their ramified processes as demonstrated by an increased microglial Iba-1 expression per cell (*p* = 0.001) and area (*p* = 0.0007) (Fig. 2g, h). Although increased, there was no significant difference in branch length(*p* = 0.1055) or perimeter/cell (*p* = 0.1711) in the stem cell group compared to the microglia of the vehicle group. Representative images of microglia from histological sections of cortical regions of all experimental groups are displayed in (Fig. 2e). Branch length(uM) [BF ctrl:176.7(90.7–197.4)]; [NEC + PBS: 27.7(14.8–60.4)]; [NEC + USC:51.8(34.6–98.7)]; Branch number [BF ctrl:55(38–73)]; [NEC + PBS: 14(7–20)]; [NEC + USC:24(16–49)]; Branch Junctions [BF Ctrl:25(19–50)]; [NEC + PBS:10 (8–18)]; [NEC + USC:27(21–31)]; Branch Endpoints [BF Ctrl:46(32–57)]; [NEC + PBS: 14(9–18)]; [NEC + USC:30(21–34)]; Iba-1 expression/cell[BF Ctrl:222.4(191.8–284.5)]; [NEC + PBS: 127.3(109.1–157.2)]; [NEC + USC:219.6(186.7–242.3)]; Span Ratio [BF Ctrl:1.78(1.22–2.52)]; [NEC + PBS: 2.06(1.75–3.07)]; [NEC + USC:1.76(1.44–2.06)]; Area (uM^2^)[BF ctrl:89,995(45,153–138,994)]; [NEC + PBS:20,111(11,236–32,249)]; [NEC + USC:40,652(25,585–57,114)]; Perimeter(uM) [BF ctrl:1,276(921–1,530)]; [NEC + PBS: 641.5(487.1–800.8)]; [NEC + USC:846(662.6–1036)].

### Stem cell therapy improves lung injury

**Fig. 3:**
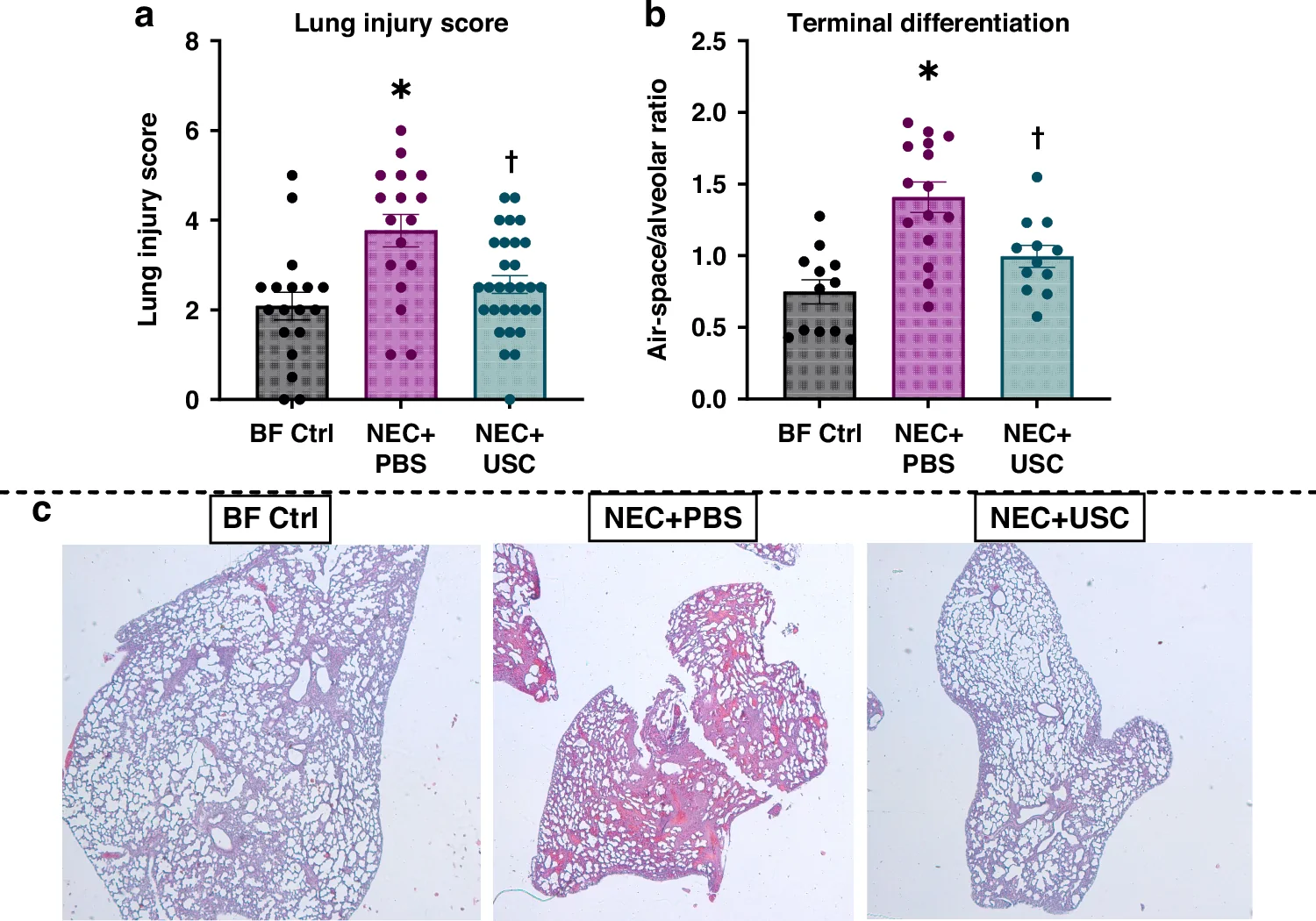
Stem cell rescue therapy prevents acute lung injury. Mice of the vehicle group (NEC + PBS) show (**a**) increased lung injury compared to both breast-fed controls (*p* = 0.0004) and stem cell (NEC + USC) group ( *p*= 0.0052). This pattern persists with the vehicle group showing worse (**b**) terminal differentiation (higher airspace/alveolar ratio) compared to both breast-fed controls (*p* = 0.0003) and stem cell (NEC + USC) group (*p* = 0.0402). Represented images are shown of lungs in each experimental group. (**c**) Representative histologic images of the lungs of breast-fed controls (BF Ctrl), vehicle group (NEC + PBS), and the stem cell therapy group (NEC + USC) are shown with scale bars reflecting a distance of 100 µM. (*p < 0.05) Comparisons between breast-fed controls (BF ctrl) and vehicle controls (NEC + PBS), (†*p* < 0.05) comparisons between vehicle group (NEC + PBS) and stem cell therapy groups (NEC + USC).

Upon examination of the captured images, the lungs of mice in the vehicle (NEC + PBS) group showed increased lung injury scores (*p* = 0.0004) when compared to the lungs of breast-fed controls which is ameliorated (*p* = 0.0052) in the stem cell therapy (NEC + USC) group [BF ctrl: 2.083 ± 0.309; NEC + PBS: 3.765 ± 0.362; NEC + USC: 2.567 ± 0.199] (Fig. 3a). When looking at terminal differentiation, the alveoli of mice in the breast-fed controls had a lower air space/alveolar ratio indicating better terminal differentiation of alveoli in contrast to the vehicle group (*p* = 0.0003). Furthermore, mice in the stem cell treatment group (NEC + USC) also had lungs with a lower air space/alveolar ratio(*p* = 0.0402) (Fig. 3b) when compared to the vehicle group, akin to the breast-fed controls. Representative images (Fig. 3c) from the whole lung sections show breast-fed controls (BF Ctrl)with well-differentiated terminal alveolar sacs and minimal hemorrhage/injury, while those of the vehicle group (NEC + PBS) show poorer terminal differentiation and more alveolar hemorrhage/signs of injury. The lungs of the therapy group (NEC + USC) mirror those of the breast-fed controls.

### Stem cell therapy improves cytokine profile in the lung/intestines but not in serum samples

**Fig. 4:**
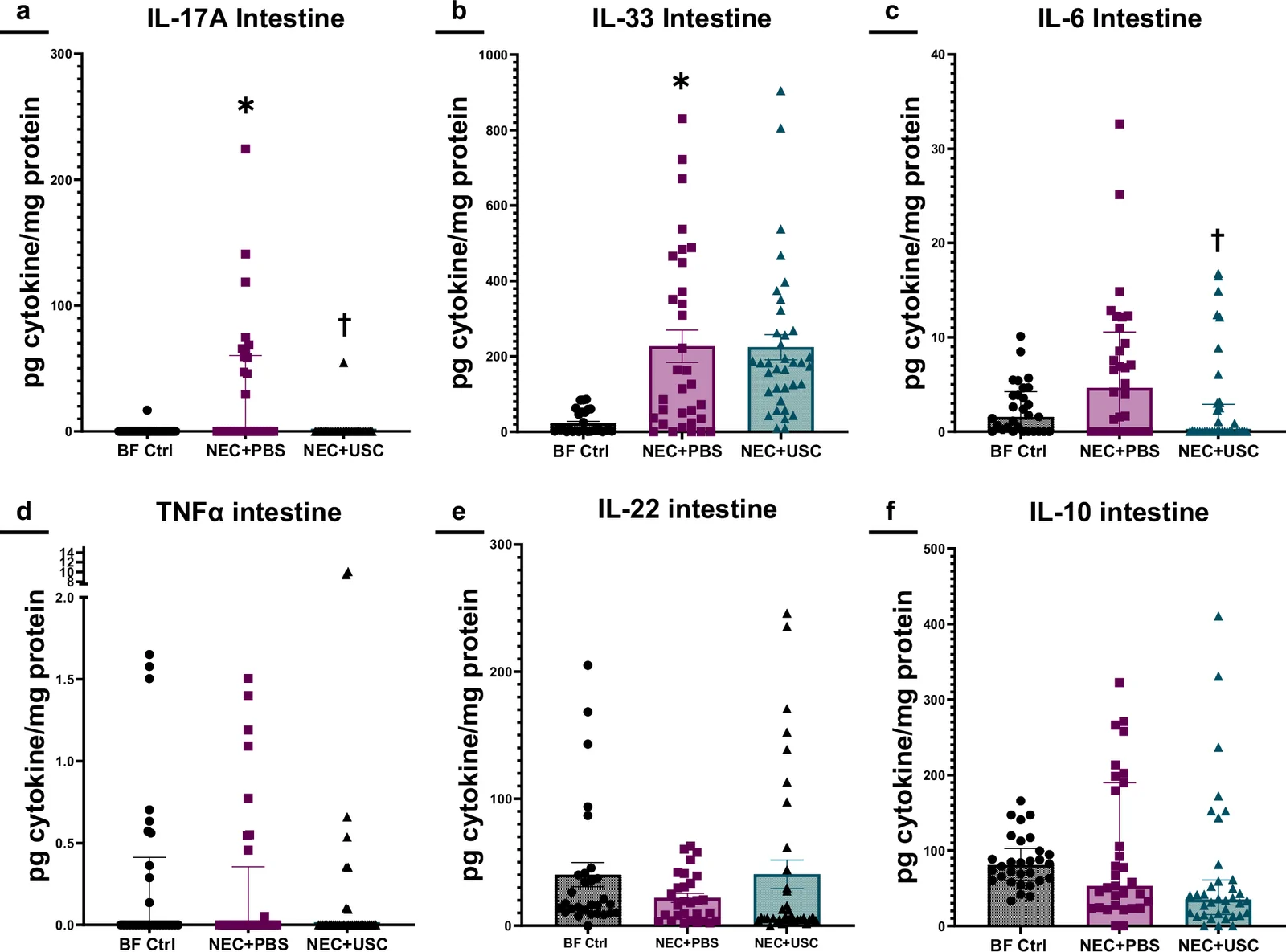
Stem cell rescue therapy improves the cytokine profile of intestines. In the intestine, the vehicle group (NEC + PBS) had significantly more (**a**) IL-17A (*p* < 0.0001) and (**b**) IL-33 (*p* < 0.0001) compared with breastfed controls, and there was a significant decrease in IL-17A (*p* < 0.0001) and (**c**) IL-6 (*p* = 0.0125) in the stem cell therapy (NEC + USC) group compared to the vehicle group. (*p < 0.05) Comparisons between breast-fed controls (BF ctrl) and vehicle controls (NEC + PBS), (†*p* < 0.05) comparisons between vehicle group (NEC + PBS) and stem cell therapy groups (NEC + USC).

In the intestine, there were significantly elevated levels of IL-17A (*p* < 0.0001) and IL-33 (*p* < 0.0001) in the vehicle group (NEC + PBS: *n* = 32) when compared to breast-fed controls (BF ctrl: *n* = 30). In the stem-cell therapy group (NEC + USC: *n* = 36), there was a significant decrease in proinflammatory cytokine of IL-17A (*p* < 0.0001) and IL-6 (*p* = 0.0125) (Fig. 4a–c). There were no differences between groups in cytokine levels of IL-10, TNF-α and IL-22 (Fig. 4d–f). [IL-17A (pg cytokine/mg protein) BF ctrl: 0(0–0); NEC + PBS: 0(0–60.26); NEC + USC: 0(0–0)]; [IL-33 (pg cytokine/mg protein) BF ctrl: 5.049(1.705–51.70); NEC + PBS: 120.5(26.55–429.6); NEC + USC: 182.8(108.9–266.5)] [IL-10 (pg cytokine/mg protein) BF ctrl: 80.64(60.1–102.8); NEC + PBS: 53.15(24.37–189.9); NEC + USC: 34.93(15.02–60.78)]; [IL-6 (pg cytokine/mg protein) BF ctrl: 1.557(0–4.251); NEC + PBS: 4.649(0–10.57); NEC + USC: 0(0–2.905)] [TNF-α (pg cytokine/mg protein) BF ctrl: 0(0–0.4126); NEC + PBS: 0(0–0.356); NEC + USC: 0(0–0)]; [IL-22 pgcytokine/mg protein BF ctrl: 16.93(10.56–40.62); NEC + PBS: 18.12(4.025–36.16); NEC + USC: 6.308(4.693–40.45)].

**Fig. 5:**
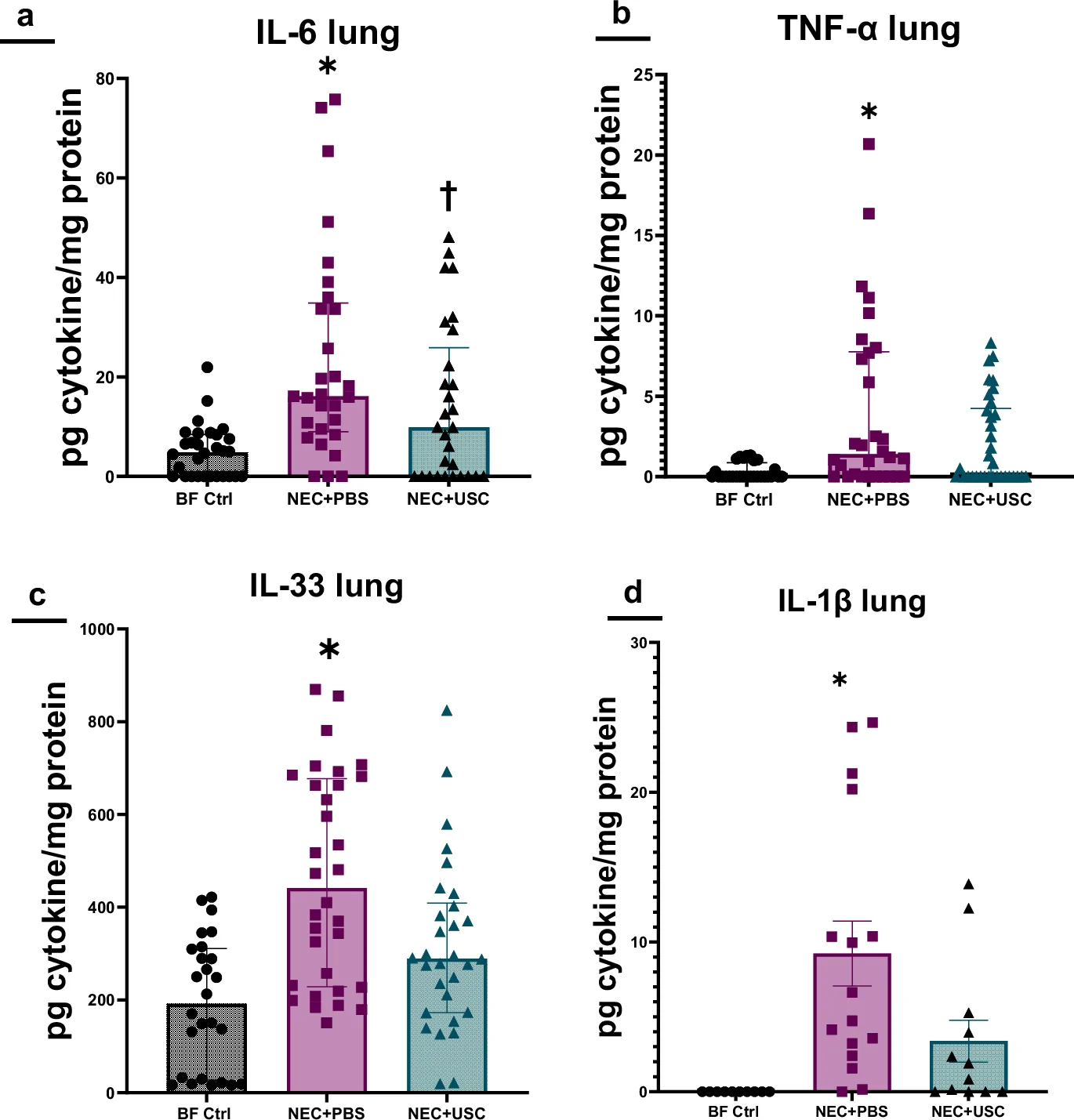
Stem cell rescue therapy improves the cytokine profile of lungs. In the lungs, the vehicle group (NEC + PBS) had significantly elevated (**a**) IL-6 (*p* < 0.0001) (**b**) TNF-α (*p* = 0.0005), (**c**) IL-33(*p* < 0.0001), and (**d)** IL-1β (*p* < 0.0001) compared with breast-fed controls. There was a significant decrease in (**a**) IL-6 (*p* = 0.0481) in the stem cell therapy (NEC + USC) group compared to the vehicle group. (*p < 0.05) Comparisons between breast-fed controls (BF ctrl) and vehicle controls (NEC + PBS), (†*p* < 0.05) comparisons between vehicle group (NEC + PBS) and stem cell therapy groups (NEC + USC).

In the lungs, there was a significantly elevated level of IL-6 (p < 0.0001), TNF-α (*p* = 0.0005), IL-33 (*p* < 0.0001), and IL-1β (*p* < 0.0001) in the vehicle group (NEC + PBS: *n* = 32) when compared to breast-fed controls (BF ctrl: *n* = 30). In the stem-cell therapy group (NEC + USC: *n* = 36), there was a significant decrease in proinflammatory cytokine of IL-6 (*p* = 0.0481), but no significant decrease of TNF-α(0.2187), IL-33 (*p* = 0.051), and IL-1β (*p* = 0.2897) (Fig. 5a–d). [IL-6 (pg cytokine/mg protein) BF ctrl: 4.83(0–8.46); NEC + PBS: 16.14(8.97–34.86); NEC + USC: 9.91(0–25.90)]; [TNF-α (pg cytokine/mg protein) BF ctrl: 0(0–0.88); NEC + PBS: 1.39(0–7.77); NEC + USC: 0.25(0–4.24)] [IL-33 (pg cytokine/mg protein) BF ctrl: 191.5(27.5–310.9); NEC + PBS: 441.3(228.5–677.2); NEC + USC: 289.1(172.8–409.1)]; [IL-1β (pg cytokine/mg protein) BF ctrl: 0(0–0); NEC + PBS: 5.68(2.61–17.75); NEC + USC: 1.37(0–4.94)].

**Fig. 6:**
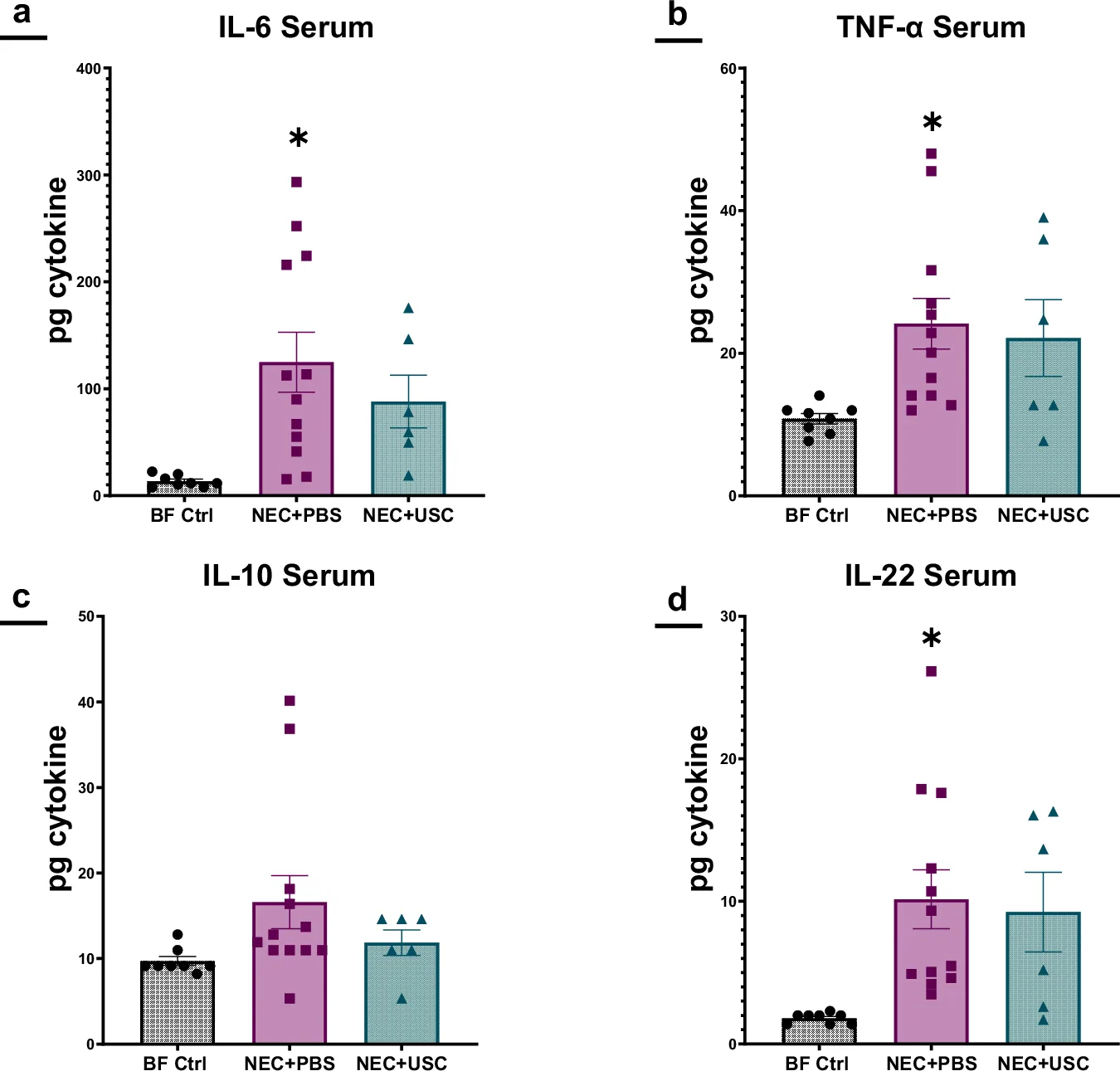
Stem cell rescue therapy does not impact the cytokine profile in the serum. In the serum the vehicle group (NEC + PBS) had significantly elevated level of (**a**) IL-6 (*p* < 0.0054), (**b)** TNF-α (*p* = 0.0213), and (**d**) IL-22 (*p* = 0.0005) compared with breast fed controls with no significant decrease in cytokine level in the stem-cell therapy groups. **d** There were no differences in IL-10. (*p < 0.05) Comparisons between breast-fed controls (BF ctrl) and vehicle controls (NEC + PBS), (†*p* < 0.05) comparisons between vehicle group (NEC + PBS) and stem cell therapy groups (NEC + USC).

In serum, there was a significantly elevated level of IL-6 (*p* < 0.0054), TNF-α (*p* = 0.0213), and IL-22 (*p* = 0.0005) in the vehicle group (NEC + PBS: *n* = 12) when compared to breast-fed controls (BF ctrl: *n* = 8). In the stem-cell therapy group (NEC + USC: *n* = 6), there was no significant decrease of IL-6 (*p* = 0.5396), TNF-α (0.9132), IL-22 (*p* > 0.9999). There were no significant differences in IL-10 (Fig. 6a–d)*.* [IL-6 (pgcytokine/mg protein) BF ctrl: 13.55 ± 1.93; NEC + PBS: 124.9 ± 27.93; NEC + USC: 88.10 ± 24.65] [TNF-α (pg cytokine/mg protein) BF ctrl:10.81 ± 0.73; NEC + PBS: 24.16 ± 3.54; NEC + USC: 22.15 ± 5.34] [IL-22 (pg cytokine/mg protein) BF ctrl: 2(1.29–2); NEC + PBS: 7.41(4.68–16.29); NEC + USC: 9.43(2.38–16.11)]; [IL-10 (pg cytokine/mg protein) BF ctrl: 10.53(9.14–10.53); NEC + PBS: 12.36(10.99–17.71); NEC + USC: 12.80(9.58–14.61)].

## Discussion

 NEC remains a devastating, morbid disease affecting young neonates. Our findings align with previous studies demonstrating the potential of mesenchymal stem cell therapy in the treatment of NEC via an intraperitoneal mechanism. Specifically, our studies show for the first time, in a NEC model, that umbilical-cord-derived mesenchymal stem cell therapy can be used as a rescue therapy after the onset of the NEC experimental protocol. Specifically, we demonstrate that a single rescue dose of USC therapy (given halfway through the NEC experimental protocol) curtails the clinical deterioration and the development of deleterious pathological changes in the intestinal, pulmonary, and cerebral architecture.

Our study reinforces that USC therapy can mitigate intestinal injury and suppress local pro-inflammatory cytokines and improve clinical outcomes, which is in line with current literature. The literature demonstrates that intestinal improvement is characterized by suppressed inflammatory response in the intestine, anti-apoptotic properties, improved tight-junction integrity, and limited bacterial translocation across the gut barrier.^6,53–55^ Our study shows that treatment with local MSCs improves the cytokine profile of the intestine to mitigate the increase IL-17A and IL-6 (both of which are pro-inflammatory cytokines).

Beyond the intestinal tract, NEC and the associated systemic inflammatory response associated with NEC contributes to neurodevelopmental and pulmonary insults.^29^ Our findings highlight the potential of USC therapy in mitigating injury to these secondary organs. Specifically, we demonstrate an improvement in microglial morphology with the treatment of umbilical-cord-derived MSCs. In the lungs, we demonstrate an improvement in lung histology and terminal differentiation—a marker of normal pulmonary and alveolar development. The cytokine profile in the lungs also demonstrated a significant improvement in IL-6 release—a potent proinflammatory cytokine—with treatment with USCs.

An understanding of the mechanism of how USC therapy mitigates this disease process is still needed. We attempted to see if local USC therapy improved the systemic signaling of proinflammatory cytokines within the serum. However, while the disease state results in an elevation of IL-6, TNF-α, and IL-22 in the serum, IP USC therapy doesn’t, in turn, propagate a decrease of this signaling in the serum.

IL-6, TNF-α, and IL-1β are acute-phase reactants that are activated early in NEC. IL-6 is cytokine that helps to drive T-helper 17 cell differentiation, resulting in an increase of IL-17A, which is a cytokine that disrupts tight junctions and recruits neutrophils.^56,57^ Our study supports that these markers are important markers in the inflammatory signaling cascade, as they are elevated in the intestine and lungs of the disease state in our mice. Although the serum cytokine levels of the disease state (NEC + PBS) reflect an increased inflammatory cell/immune cell activation profile, there is no significant improvement with USC therapy. This does support the theory that USC therapy acts locally, and it could act via a paracrine mechanism,^6,38^ which is the current prevailing theory. The inflammatory cascade triggered by the intestinal insults that is characteristic of NEC results in activation of the IL-6/IL-17A axis, which is well characterized in the literature.^56,58–60^ This in turn could result in an inflammatory reaction of the brain and lungs through a yet to be elucidated connection of the brain-gut-lung axis.^27,29,61^ The mechanism of IP USC therapy in NEC may be to mitigate the effects of the IL-6/IL-17A intestinal inflammatory cascade, as seen by their significant local reduction in our data with clinically improved outcomes and improved intestinal histological appearance in our stem cell therapy group. Because of the reduced inflammatory signaling within the intestine, there may be less gut-lung crosstalk, which results in a fall of lung-IL-6 secretion, leading to a dampened inflammatory signaling response in the lungs, as seen in our data. This suspected decrease in inflammatory signaling from the gut could be why, in the lungs, we clinically observe improved alveolarization and protection from the bronchopulmonary dysplasia-like changes as seen in our NEC experimental model.

This gut-anchored, paracrine-first mechanism is our proposed hypothesis, which elegantly explains why USC therapy alters *local* intestinal (IL6↓, IL17A↓) and *distal* pulmonary (IL6↓) cytokine landscapes while leaving systemic TNFα and other signaling molecules largely unchanged. Understanding these intricacies is the future of our work.

Despite these promising results, our study has limitations. The murine model does not serve as a perfect example of the complexity of NEC in human neonates and will warrant further studies on dosing/timing as well as safety profile of USC administration. It can be difficult to attribute the microglial/lung changes solely to intestinal inflammation when key components of the NEC protocol include hypoxia. Regardless, we have demonstrated clinical/histological improvement with local intestinal USC therapy, which at least hints at a gut-brain and gut-lung axis.

Our experimental design also has several limitations. For the purposes of this experiment, a mix of male and female mice was used without stratifying on sex-based differences. Our rationale is that mice have low levels of circulating hormone until several weeks after birth. Additionally, we did not include a baseline assessment of clinical and histological injury on day 7 of life prior to treatment with USCs. In our study, by day 7, our mice were showing signs of clinical injury, with many murine models of NEC also showing clinical and histological changes by the first two days of life.^41,42,44–46^ Additionally, our cytokine data reflect the inflammatory response in mice of a murine model of NEC on day 9 of life (4 days after onset of the inflammatory trigger). This could explain the lack of cytokine response of earlier inflammatory phase cytokines (including TNF-α and IL-1β) in our experiment. Future studies should look at the inflammatory response at each day of the experimental design to look at how the immune landscape changes. Additionally, our total “n” for our serum samples is lower than our tissue samples as we didn’t began harvesting serum until later runs of our experimental protocol, which may have underpowered our results in this compartmental analysis. Finally, while our findings suggest the role of USCs in reducing NEC-associated neuroinflammation and lung injury, further understanding of the mechanism behind these effects is necessary. This could lend itself to the study of stem cell alternatives, including exosomes, or isolating the beneficial aspects of USC therapy to package the therapy into a safer alternate.

Several of these limitations can be addressed with the use of larger animal models—such as a piglet model, which has an NEC induction in a remarkably similar fashion to premature neonates. Another purpose of exploring larger models is to look at the risks associated with USC therapy, including the theoretical risks of engraftment, coagulopathy, and aberrant differentiation. The additional benefit of larger animal models is to also explore alternate therapeutic delivery methods. Because of these risks, prior to initiation of translational applications, the process to transition to clinical trial testing will be rigorous.

## Conclusion

In conclusion, our findings suggest that USCs can be effectively utilized as a rescue therapy and can even mitigate insult to secondary organ systems such as the lung and the brain. This further broadens their clinical applicability in neonates diagnosed with NEC. Further research into the mechanism and safety profile of this treatment is needed to promote the clinical availability of this treatment.

## Supplementary information


Supplementary dataset


## Data Availability

The cytokine data is available as a Supplementary dataset. The datasets generated during and/or analyzed during the current study are available from the corresponding author on reasonable request.
